# Inferring Reputation Promotes the Evolution of Cooperation in Spatial Social Dilemma Games

**DOI:** 10.1371/journal.pone.0040218

**Published:** 2012-07-09

**Authors:** Zhen Wang, Lin Wang, Zi-Yu Yin, Cheng-Yi Xia

**Affiliations:** 1 Key Laboratory of Computer Vision and System (Ministry of Education) and Tianjin Key Laboratory of Intelligence Computing and Novel Software Technology, Tianjin University of Technology, Tianjin, China; 2 School of Physics, Nankai University, Tianjin, China; 3 Department of Physics, Hong Kong Baptist University, Kowloon Tong, Hong Kong; 4 Center for Nonlinear Studies and the Beijing-Hong Kong-Singapore Joint Center for Nonlinear and Complex Systems (Hong Kong), Hong Kong Baptist University, Kowloon Tong, Hong Kong; 5 Adaptive Networks and Control Lab, Department of Electronic Engineering, Fudan University, Shanghai, China; Universidad Carlos III de Madrid, Spain

## Abstract

In realistic world individuals with high reputation are more likely to influence the collective behaviors. Due to the cost and error of information dissemination, however, it is unreasonable to assign each individual with a complete cognitive power, which means that not everyone can accurately realize others’ reputation situation. Here we introduce the mechanism of inferring reputation into the selection of potential strategy sources to explore the evolution of cooperation. Before the game each player is assigned with a randomly distributed parameter *p* denoting his ability to infer the reputation of others. The parameter *p* of each individual is kept constant during the game. The value of *p* indicates that the neighbor possessing highest reputation is chosen with the probability *p* and randomly choosing an opponent is left with the probability 1−*p*. We find that this novel mechanism can be seen as an universally applicable promoter of cooperation, which works on various interaction networks and in different types of evolutionary game. Of particular interest is the fact that, in the early stages of evolutionary process, cooperators with high reputation who are easily regarded as the potential strategy donors can quickly lead to the formation of extremely robust clusters of cooperators that are impervious to defector attacks. These clusters eventually help cooperators reach their undisputed dominance, which transcends what can be warranted by the spatial reciprocity alone. Moreover, we provide complete phase diagrams to depict the impact of uncertainty in strategy adoptions and conclude that the effective interaction topology structure may be altered under such a mechanism. When the estimation of reputation is extended, we also show that the moderate value of evaluation factor enables cooperation to thrive best. We thus present a viable method of understanding the ubiquitous cooperative behaviors in nature and hope that it will inspire further studies to resolve social dilemmas.

## Introduction

The emergence and maintenance of cooperation through natural selection is an enduring conundrum in evolutionary biology and other related disciplines [1]. According to the Darwinian evolutionary theory [2], any behavior that contributes benefits to others but not directly to oneself will soon disappear. However, this is not fully consistent with the ubiquitous existence of cooperative behaviors in uncountable biological or social settings, especially in animal and human societies [3]–[6]. In order to solve this puzzle, a variety of game theoretical models inspired by different biological situations, such as the prisoner’s dilemma game, the snowdrift game and public goods games, have been extensively studied [7]–[18]. Most notably, the prisoner’s dilemma game has received particular renown and becomes the leading paradigm to explore the evolution of cooperation among selfish individuals [19]–[25].

As a metaphor, the prisoner’s dilemma game is often employed to investigate how the cooperation evolves between pairwise interactions, and various extensions of this model have also been proposed to further understand the origin of cooperation [19], [26]–[36]. In the original model, two players must simultaneously decide to either cooperate (*C*) or defect (*D*) without knowing the co-player’s decision, and the corresponding payoff matrix can be described as follows,
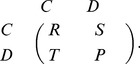
(1)


Here, both players will receive the reward *R* if they cooperate and the punishment *P* if they select non-cooperation. But when a defector meets a cooperator, he exploits the cooperator and receives the temptation *T*, and the cooperator is left with the sucker’s payoff *S*. Importantly, these terminological terms are required to satisfy the following ranking:
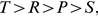
(2)and
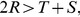
(3)from where it is clear that players need to defect if they wish to maximize their own payoffs, irrespective of the opponent’s decision. Resulting is a social dilemma, which typically leads to widespread defection.

During the past decades, various specific mechanisms have been proposed and experimentally verified to avert this unfavorable outcome of social dilemmas and to promote the evolution of cooperation among unrelated individuals. Typical examples include kin selection [37], direct and indirect reciprocity [25], [38]–[40], differences in evolutionary time scales [41], the potential influence of noise [42], [43], group selection [44], altruistic punishment [45], [46], effective strategies [47]–[50] such as the tit-for-tat or win-stay-lose-shift, and spatially structured populations [26], [51], [52]. Particularly, the spatial reciprocity has been identified as one of the most fruitful means to largely enhance the cooperation levels. For instance, when players are arranged on a lattice and interact only with their nearest neighbors, cooperators can easily survive through forming compact clusters, which rescue the cooperators from the exploitation of defectors [26]. Following this pioneering work, a great deal of modified versions and various underlying promoting mechanisms have been studied. To name but only a few, many works attest to the fact that complex networks with the connectivity structure similar to that of real-world networking systems can greatly facilitate the emergence of cooperation [19], [35], [41], [53]–[58]. The mobility of agents, if appropriately tuned, also results in the prevalence of cooperation even under the noisy conditions that do not necessarily support the diffusion of cooperators [59]–[60]. It is promising, furthermore, that the individual heterogeneity or social diversity can have a positive impact on the cooperative behaviors even if the defection temptation is large [22], [44], [64], [65] (for comparison and better review, we refer to some comprehensive works [47], [66]–[68]).

Besides the above scenarios, a new and potent approach, namely, reputation mechanism, is gaining more momentum recently [38], [69]–[73]. Turning to experimental study, Nowak and Sigmund not only unraveled that reputation by itself exerted a strong influence on cooperation dynamics, but also helped explain the high cooperation levels in human society, especially under the framework of indirect reciprocity [38]. Inspired by this delightful achievement, Fu *et al.* investigated the effect of reputation on the individual partner-switching process. They found that, if players were able to alter their behavioral strategies and their social interaction partnerships according to the reputation situation, cooperation would prevail [74]. However, reputation is not a real entity. People do not have tags on their back, which can directly illuminate their true reputation. In previous works, it is usual assumed that each person can correctly realize others’ reputation. Due to the cost and error of information dissemination, this assumption is only the ideal case. More realistic scenario will acknowledge that each individual has limited and different ability to infer others’ reputation.

Here we propose an approach that takes into account the influence of individual inferring ability during the process of reputation judgement (for simplicity, it is named referring reputation mechanism). Considering difference of the inferring ability among agents, we utilize a randomly distributed parameter 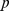 in the interval between zero and one for each player to denote his inferring ability. The value of 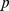 means that the neighbor possessing highest reputation is chosen with the probability 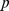 and the case of randomly choosing opponent is left with the probability 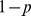. (Although the situation that each person possesses the same 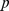 value is not studied, two extreme cases are worth being mentioned. Setting 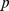 equal to zero for all the individuals returns to the traditional version, which is usually employed to measure whether cooperation is promoted under the proposed mechanism. While 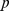 being one within the whole population is not realistic, which is incompatible with the fundamental assumption that each person has limited and different estimation ability, and spontaneously losses the meaning and necessity of comparing with our results.) Through scientific computer simulations we demonstrate, compared with the traditionally spatial version, that this simple mechanism promotes the evolution of cooperation significantly. We provide a reasonable explanation for the observed phenomenon and explore the impact of different uncertainty levels in strategy adoptions. In addition, we introduce an evaluation factor into the calculation of reputation and show the existence of optimal cooperation levels. Finally, we conclude that referring reputation outlines a viable route to resolve social dilemmas, which will inspire further studies.

## Results

### 0.1 Impact on the Evolution of Cooperation

**Figure 1:**
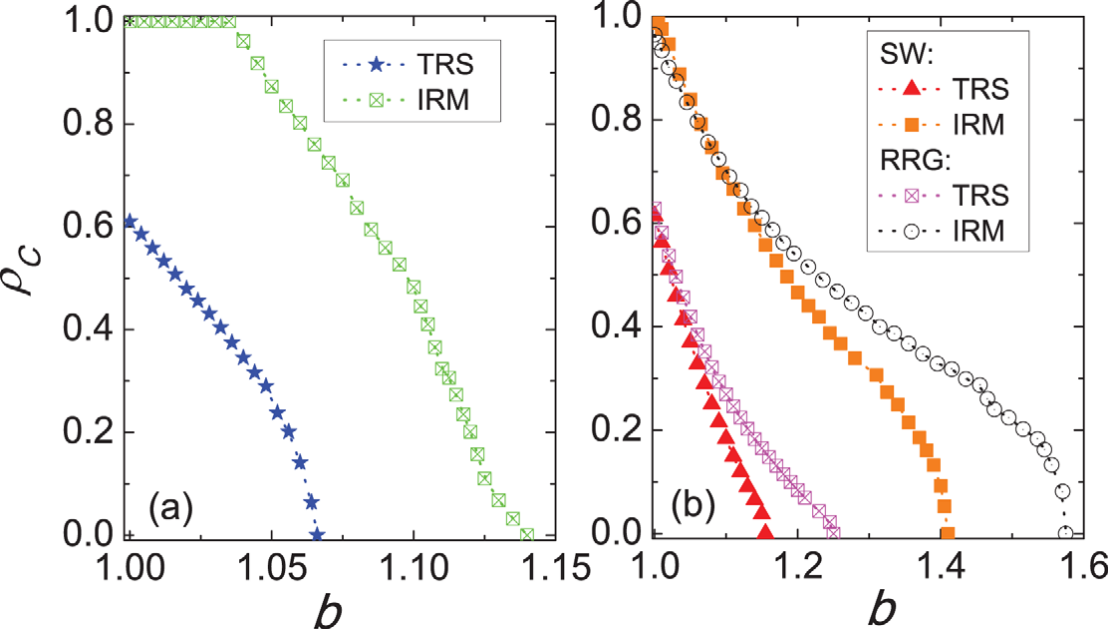
Promotion of cooperation due to inferring reputation on different networks. (a) depicts the fraction of cooperators 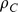 in dependence on the temptation to defect 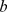 for the traditionally spatial version (TRS) and inferring reputation mechanism (IRM) where a random distribution of 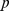 in the interval between zero and one is implemented on the square lattice. It can be observed, compared with the results of traditional version, that the novel mechanism not only enables cooperators to reach their exclusive dominance, but also allows for cooperative behaviors to prevail at high temptation to defect. (b) depicts the fraction of cooperators 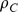 in dependence on the parameter 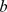 on the random regular graph (RRG) and Watts-Strogatz small-world (SW) network with the fraction of rewired links equalling 0.1. These results are in qualitatively agreement with the observations on the square lattice, supporting the conclusion that inferring reputation remarkably promotes the evolution of cooperation, irrespective of the underlying interaction networks.

As is well known, in the traditional prisoner’s dilemma game cooperators will be decimated fast even if the temptation to defect is not very high [24], [54], [75]. It thus becomes challenging to identify whether referring reputation mechanism supports the evolution of cooperation for high temptation. In order to address this puzzle, we present in Fig. 1 the fraction of cooperators 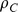 in dependence on the temptation to defect 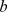 for different scenarios. It is evident, compared with the traditional version of the game, that the consideration of such a mechanism can significantly sustain the emergence and evolution of cooperation, which is consistent with our expectation [see Fig. 1(a)]. In the traditional spatial version, cooperators can exist at substantial levels if 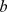 is relatively small, and then becomes less and less resilient to the invasion of defectors with the fast enhancement of 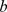 value. However, with the novel mechanism, cooperators are not only able to reach an exclusive dominance, but even prevail over a larger interval of 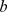. Another important but more subtle alteration is the critical threshold value 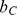, marking the extinction of cooperators. One can find that the value of 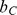 is enhanced from 1.066 to 1.14, when the judgement of reputation is introduced. These results suggest that the mechanism of inferring reputation can substantially improve the cooperation.

Strikingly, qualitatively identical results can be obtained on complex interaction networks other than the square lattice. Results presented in Fig. 1(b) depict how cooperators fare on the small-world network and the random regular graph. Similarly as in Fig. 1(a), it can be observed, compared with the results of traditional version, that cooperators perform significantly better under the novel mechanism and survive at larger values of 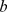. This is in agreement with the observations made on the square lattice, indicating that referring reputation mechanism is universally effective in promoting the evolution of cooperation irrespective of the underlying interaction networks. In addition, since complex networks are usually identified as the potent promoters of cooperation on their own right [35], [76], [77], the promotion effect is more conspicuous.

**Figure 2:**
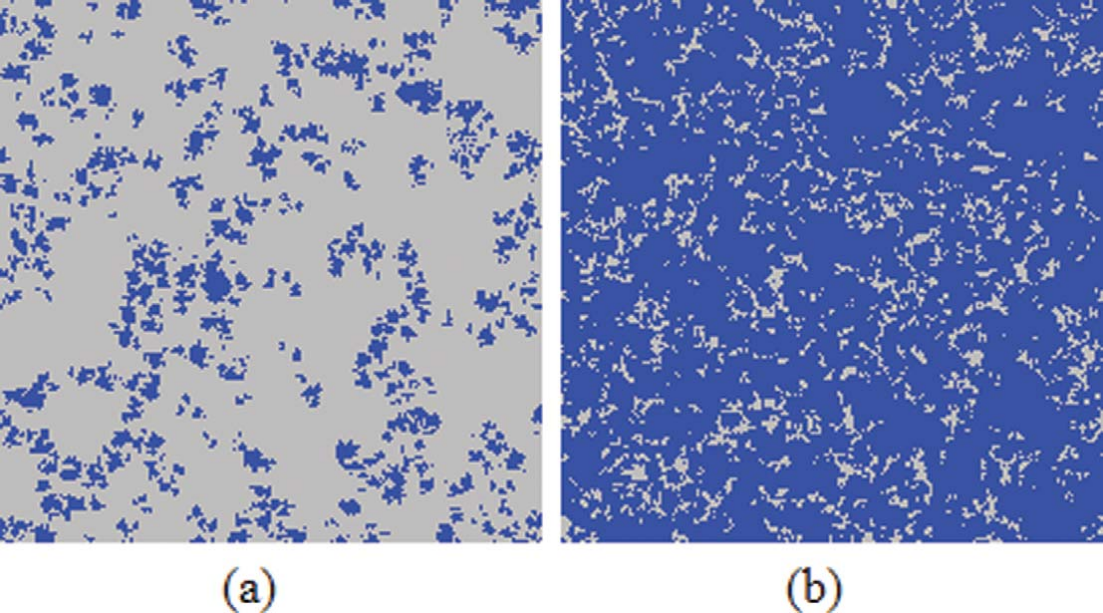
Characteristic snapshots of strategy distributions on the square lattice. (a) depicts the distributions of cooperators (blue) and defectors (gray) for the traditionally spatial version, where only a small fraction of cooperators can survive through forming the clusters to resist the defectors’ attacks. (b) depicts the distributions of players when inferring reputation is taken into account. It is obvious that the evolution of cooperation is facilitated to the point of nearly complete cooperator dominance, which is supported by the formation of extremely robust clusters of cooperators. Note that these visual observations are qualitatively consistent with results presented in Fig. 1. Depicted results were obtained for 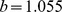.

In order to provide an intuitive assessment about the impact of the inferring reputation mechanism on the cooperative behaviors, we show in Fig. 2 the characteristic spatial distributions of cooperators and defectors for the above scenes. The result presented in Fig. 2(a) depicts the situation for the traditionally spatial version, where a small fraction of cooperators can survive on the lattice by means of forming clusters, thereby protecting themselves against the exploitation by defectors [24], [51]. Moreover, it is worth mentioning that these clusters are usually small, discrete and the distance among them is much larger than the size of clusters, which to some extent helps to explain why it is impossible to yield the absolute dominance of cooperators. Next we examine the spatial distributions of players for the novel proposed mechanism. As evidenced in Fig. 2(b), cooperators prevail even reach their undisputed dominance, whereby clustering remains their mechanism of spreading and survivability. One can find, if compared to the left snapshot, that the clusters of cooperators become larger and more compact, which further results in less space left for defectors. Importantly, this phenomenon can also be interpreted from some sociological viewpoints. For example, in economic society, the companies who always finish the production tasks or contracts with other firms on time will have higher and higher prestige. Then, more enterprises are not only willing to trade with them, but also incline to learn and imitate their way of management or technologies. As such, these illustrative snapshots attest to the fact: if players’ reputation is correlated with their cooperation behavior, it is reasonable to predict that the collective cooperation can be improved under our inferring reputation mechanism.

**Figure 3:**
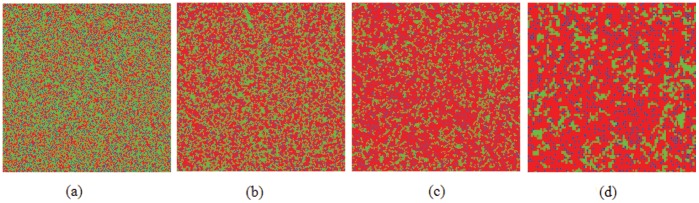
Time evolution of the clusters of cooperators on the square lattice. (a), (b) and (c) depict the distributions of individual strategies as recorded at 0, 20, 2000 steps, while (d) is an enlarged portion of (c) to show the final distributions of cooperators more distinctly. Cooperators with high (low) reputation are colored blue (red), defectors are colored green. The distributions of cooperators are initially random, but soon cooperators with high reputation help more individuals form clusters to resist defector attacks, since they are chosen more likely as the potential strategy donors. With these clusters mushrooming, less space is left to defectors. Note that cooperators with high reputation are usually located at the centers of clusters, which is beneficial for inducing the transformation from defectors to cooperators along the boundaries of clusters. Depicted results were obtained for 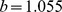.

Now that the inferring reputation mechanism enables the formation of extremely robust clusters of cooperators [see Fig. 2(b)], it is significant to explore why it can lead to larger clusters. To answer this question better, cooperators are separated into two types: cooperators with high reputation and cooperators with low reputation (we assume that, if the reputation of a cooperator is larger than that of all its neighbors, it will possess high reputation, otherwise its reputation being low). We present in Fig. 3 the evolution process of cooperator clusters, whereat defectors are colored green, cooperators with high and low reputation are colored blue and red, respectively. In the beginning [see Fig. 3(a)], the distributions of cooperators are random. As the evolution proceeds we observe that cooperators quickly shape effective clusters to resist the invasion of defectors, which is consistent with the results of traditionally spatial populations [24], [51]. However, the subsequent situation is interesting: the clusters of cooperators become larger and more compact. Strikingly, cooperators with high reputation are basically located at the centers of clusters, while cooperators with low reputation lie along the boundaries of clusters [see the enlarged snapshot in Fig. 3(d)]. We argue that cooperators with high reputation play a crucial role in sustaining the stable clusters of cooperators. Namely, during the evolution process, cooperators with high reputation will be chosen more likely as potential strategy donors, which induces more cooperators approaching and surrounding them. Consequently, the initial clusters warranted by the spatial reciprocity alone start mushrooming to depress the invasion of defectors. It is also natural that their followers, *i.e.*, cooperators with low reputation, usually lie along the boundaries. In a sea of cooperators this is practically always these followers rather than defectors trying to penetrate into the clusters. This kind of expansion ultimately results in highly robust clusters of cooperators that goes beyond the observation supported by spatial reciprocity alone [66], [78].

**Figure 4:**
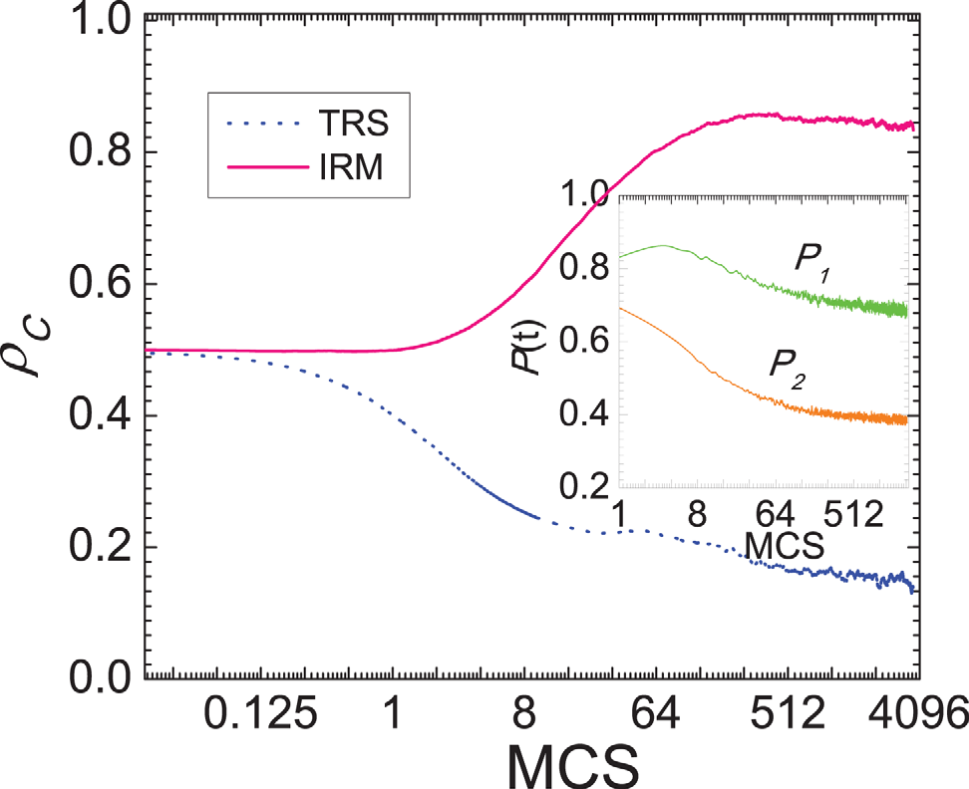
Time courses of the fraction of cooperators on the square lattice. Results are presented for the traditional version (dotted line) and inferring reputation mechanism (solid line). Evidently, when the inferring reputation is considered, the initial temporary downfall of cooperators is effectively halted and turns to their fast prevalence. During this process cooperators with high reputation play a crucial role, since they are more likely regarded as the potential strategy sources, especially in the early stages. In order to provide quantitative evidence, the inset depicts the evolutionary process of two parameters, which are closely related to the chosen frequency of cooperators with high reputation. It is obvious that the decline of 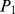 and 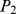 corresponds to the enhancement of cooperation. Note that the horizontal axis is logarithmic and that values of 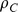 were recorded also in between full Monte Carlo steps (MCS) to ensure a proper resolution. Depicted results were obtained for 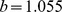.

Next, it is interesting to quantitatively elucidate why cooperative behaviors are supported under inferring reputation mechanism. To provide answers, we examine in Fig. 4 the time courses of 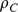 for different scenarios. What first attracts our attention is the evolution status for the traditional version, namely, in the very early stages of evolutionary process (note that values of 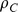 were recorded also in between full Monte Carlo steps) the performance of defectors is better than that of cooperators. This is consistent with what one would expect, given that defectors are, as individuals, more successful than cooperators and will thus be chosen more likely as potential strategy donors. This in turn amplifies their chances of spreading and ultimately result in the decimation of cooperators (only between 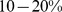 survive). However, for the proposed model with the judgement of reputation, the new situation appears: it quickly restrains from the exploitation of defectors and is in favor of the prosperity of cooperators. That is, the spatial reciprocity alone is not enough to maintain high cooperation levels after the transformation, and the inferring reputation mechanism is responsible for the emergence and maintenance of cooperation on the square lattice. In the very early stages, since cooperators with high reputation can be frequently chosen as the potential strategy donors, the advantage of defectors is weakened largely. Simultaneously, these cooperators attract more individuals to form effective clusters through leading the transformation from defectors to cooperators. Crucial thereby is the fact that the clusters built by cooperators are impervious to the lure of becoming defectors and able to recover the space of defectors, which ultimately results in widespread cooperation going beyond what can be warranted by the spatial reciprocity alone [66], [78]. After cooperators reach their utter dominance, the size of clusters will keep nearly constant, namely, the frequency that cooperators with high reputation are chosen as the potential strategy donors becomes steady. Particularly, in order to validate the above comment we also investigate the time courses of two new statistical parameters defined as follows,
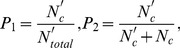
(4)where 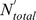 denotes the imitated number of neighbors with highest reputation during one step, 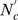 represents the number of cooperators among 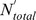, and 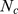 corresponds to the number of randomly chosen cooperators. Obviously, 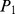 and 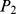 are related to the chosen frequency of cooperators with high reputation. In the early stages of evolutionary process, their values are high, which to a large extent protects more cooperators against the exploitation by defectors [see the inset of Fig. 4]. As the game proceeding the clusters formed by cooperators expand quickly, and due to the fact that cooperators located along the boundaries of clusters increase the values of 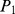, 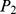 have a decline, which corresponds to the enhancement of 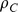. Thus, we argue that inferring reputation induces a recovery effect halting and eventually reverting the decrease of cooperation toward their absolute dominance.

**Figure 5:**
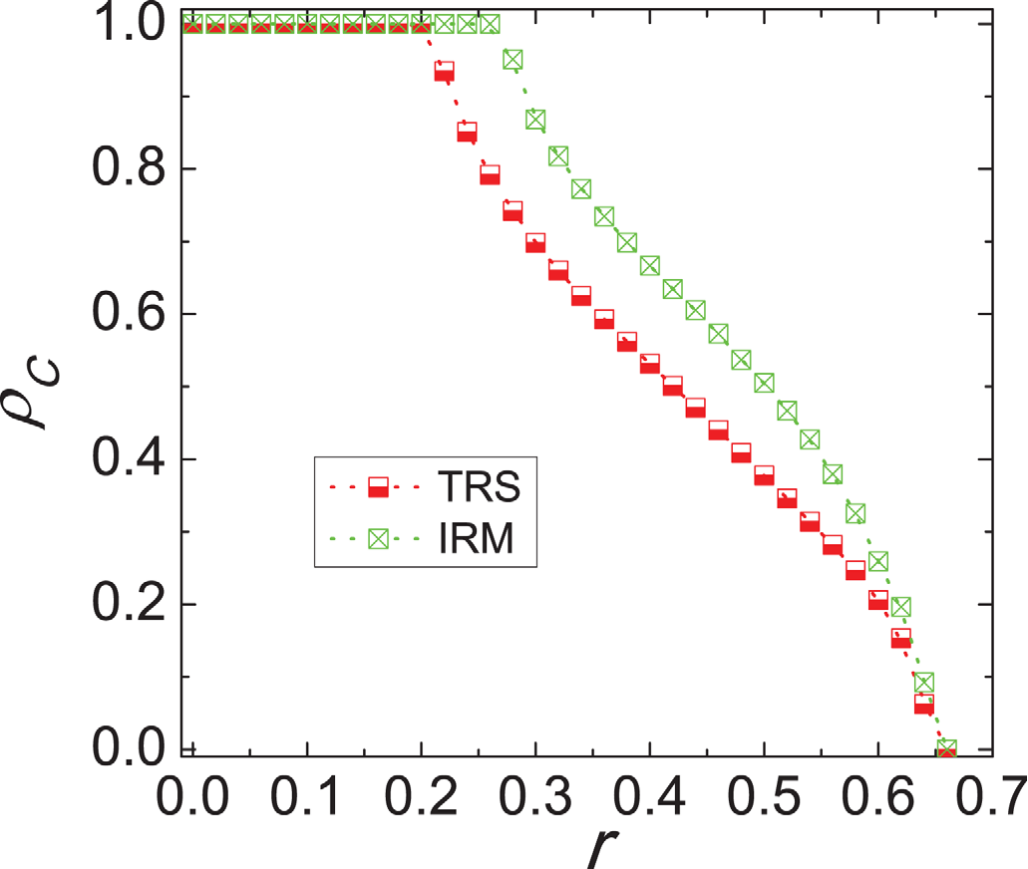
Promotion of cooperation for snowdrift game on the square lattice. This depicts the fraction of cooperators 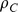 in dependence on the cost-to-benefit ratio *r* for the traditionally spatial version and the inferring reputation mechanism. Similarly as the results of prisoner’s dilemma game, it can be observed that cooperation is promoted under the novel mechanism (though the promotive effect is less remarkable), which further supports its universality.

Till now, the promotive impact of inferring reputation mechanism is merely restricted to the prisoner’s dilemma game. In order to widen its generality, it is significative to explore its effect in other evolutionary games. Due to the well-known claim that spatial structure inhibits the evolution of cooperation in the snowdrift game [34], the snowdrift game naturally becomes an appropriate candidate for this task. We present in Fig. 5 the fraction of cooperators 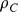 in dependence on the cost-to-benefit ratio 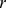 for different scenarios. Similarly as in Fig. 1, it can be observed that under the proposed mechanism the evolution of cooperation is promoted, which is qualitatively consistent with the results obtained for the prisoner’s dilemma game. It is worth noting that the promotive effect is less pronounced, which may be attributed to the fact that the spatiality is indeed less crucial for the evolution of cooperation in the snowdrift game. Nevertheless, this observation supports the fact that the newly identified mechanism facilitating the evolution of cooperation is generally valid.

### 0.2 Phase Diagram-Influence of Uncertainty *K*

**Figure 6:**
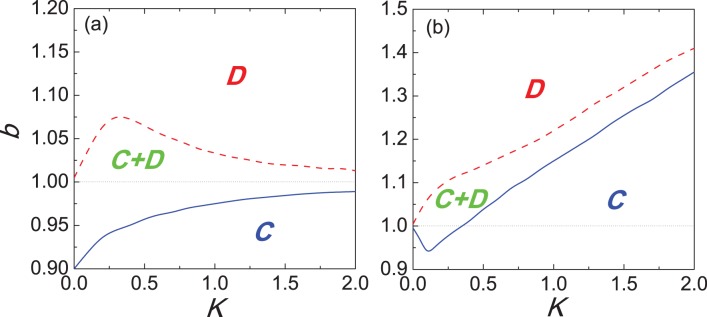
Full *b*−*K* phase diagrams on the square lattice. Blue solid and red dashed lines mark the border between pure 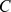 and 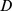 phases and the mixed 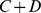 phase, respectively. (a) depicts the phase diagram for the traditional version. There exists an intermediate uncertainty in the strategy adopting process, where the survivability of cooperators is optimal, i.e., 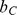 is maximal. (b) depicts the phase diagram under the inferring reputation mechanism, which is qualitatively different from the above case. In addition to the monotonous increasing borderline separating the pure 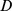 and mixed 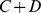 phases, a non-monotonous 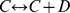 transition has also replaced the monotonous one. Since the phenomenon in (a) can only be observed on the interaction topologies lacking overlapping triangles [76], [80], the change of phase transition is attributed to the possible alternations of effective interaction networks.

An important remaining question is to examine the evolution of cooperation in dependence on the uncertainty by strategy adoptions. While the levels of uncertainty can be tuned by 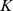 [see Eq.(7)], which acts as a temperature parameter in the employed Fermi strategy adoption function [51]. When 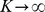 all information is lost and the strategies are adopted by means of a toss coin. We show in Fig. 6 the full 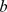
–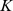 phase diagrams for different scenarios. The phase diagram in Fig. 6(a), in addition to the monotonous increasing border between the pure 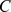 and mixed 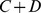 phases, features a bell shaped phase boundary separating the pure 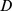 and mixed 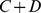 phases, implying the existence of an optimal level of uncertainty (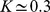) for the evolution of cooperation. This phenomenon can be interpreted as an evolutionary resonance [79], and only be observed on the interaction topologies lacking overlapping triangles [80], [81]. Interestingly, the consideration of new identified mechanism drastically changes this situation, as can be observed from the phase diagram presented in Fig. 6(b). Instead of the optimal outlay, the 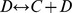 transition line is monotonically increasing towards the large 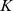 limit, which means promotive impact prevails across the whole span of 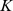. On the other hand, the lower phase boundary separating the pure 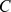 and mixed 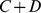 phases becomes an inverted bell-shaped line, indicating the existence of an optimal uncertainty 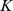 (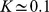) for defection. Therefore, the phase diagrams of Fig. 6 seem to indicate that for the inferring reputation mechanism, there may be a change in the effective interaction topology. The square lattice obviously lacks overlapping triangles and thus enables the observation of an optimal 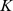 for the evolution of cooperation, while introducing the judgement of reputation into the selection of potential strategy donors makes it possible to enhance the linkage among essentially disconnected triplets, which in turn alters the evolution of cooperation. A similar phenomenon has been observed in public goods games as well [81]. It would be interesting to further investigate the structure of such effective topology.

### 0.3 Extended Version

From the definition of reputation (which is calculated in an accumulated way, referring Methods for more details), one can see that historical memory and present strategy selection jointly play an important role in the evaluation of reputation. Only if players make enormous efforts previously and at present, can they get high reputation. However, another fact also attracts great attention, namely, historical memory and current selection should have different percentage in the evaluation of individual state. A similar viewpoint has been studied in the measure of individual fitness [82]. It is, therefore, instructive to introduce an evaluation factor 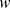 into the calculation of reputation and examine its effect on the evolution of cooperation. Then the individual reputation can be evaluated according to the following expression,
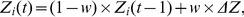
(5)where the evaluation factor 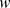 (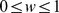) is a tunable parameter. Evidently, for 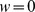 individual reputation mathematically equals the historical memory, which is not related with current situation. For 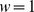 the reputation fully depends on its present strategy selection.

**Figure 7:**
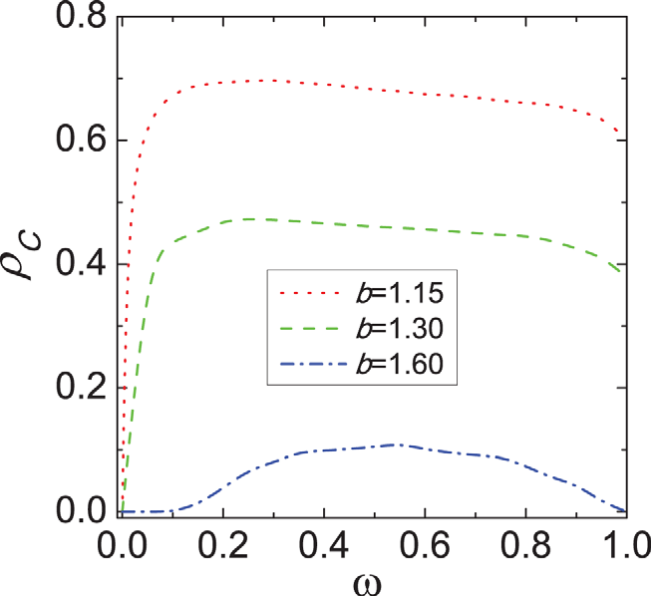
Effect of evaluation factor *w* on the evolution of cooperation. This depicts the fraction of cooperators 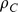 in dependence on the evaluation factor 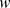 for different values of parameter 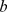. It can be observed that for the moderate value of 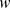 cooperators thrive best.

To explore its impact, we present in Fig. 7 the fraction of cooperators 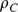 in dependence on 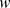 for different values of 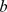. It can be observed that cooperative behaviors go through a non-monotonous change. For 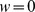 the outlook of cooperators is gloomy and they do not avoid the destiny of vanishing. However, with the increment of 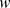 an inspiring result appears: the promotion of cooperation is really remarkable and can reach an optimal level at the moderate value of 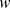. After that value, the spreading of cooperation is halted and turns to gradual decline. Hence, there exists the moderate value of 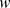 leading to the optimal evolution of cooperation.

## Discussion

In sum, we have shown that inferring reputation, *i.e.* the ability of identifying the highest reputation neighbors as potential strategy donors, may be seen as an universally applicable promoter of cooperation irrespective of the underlying interaction networks and evolutionary games. By means of extensive simulations, we have found that cooperators with high reputation play a crucial role in the evolution. They not only induce a collective resistance against the invasion of defectors, but importantly accelerate the formation of extremely robust clusters of cooperators, whereat they are more likely to be regarded as the potential strategy donors and surrounded by more followers. Another interesting finding is that the consideration of such a simple yet meaningful mechanism effectively alters the interaction networks. In its absence there exists an intermediate uncertainty 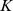 where cooperators can survive at the largest temptation of defect, while in its presence this feature vanishes and becomes more promotive for the cooperators (especially noting the boosted boundary line between pure 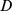 and mixed 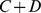 phases). However, since the actual topology structure always remains unaffected, we have attributed the differences in the evolution of cooperation to the possible alternation of the effective interaction topology, which is brought about by the fact that cooperators with high reputation are more likely to act as the sources of adopted strategies. Therefore, the connections between previously unrelated individuals seem to become stronger than average. Lastly, by studying an extended version where the calculation of individual reputation involves the evaluation factor, we have shown that a moderate value of evaluation factor enables the observation of an optimal cooperation level.

Evidently, the inferring reputation seems widely applicable as well justifiable with realistic examples. For example, some distinguished talents in general have high reputation and they are more likely to affect the collective behaviors than others. However, under certain situations, it is also possible that individuals can not correctly identify their circumstances due to the cost and error of information dissemination. In this sense, the random selection of a partner becomes a most frequently adopted alternative. Since this work appears very reasonable, we hope that it will inspire further studies, especially in terms of the solution of some social puzzles via a coevolutionary process [67]. Moreover, we can also evaluate individual reputation from other sociological viewpoints. For example, unlike the independent treatment in the present work, reputation can be regraded as a concept consequence of the opinion from the rest of population.

## Methods

We consider an evolutionary prisoner’s dilemma game that is characterized with the temptation to defect 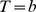 (the highest payoff received by a defector if playing against a cooperator), reward for mutual cooperation 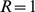, the punishment for mutual defection 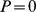, and the sucker’s payoff 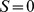 (the lowest payoff received by a cooperator if playing against a defector), whereby 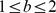 ensures a proper payoff ranking. Although being in effect the so-called weak prisoner’s dilemma in that 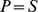 rather than 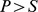, this version captures all the relevant aspects of the game [26]. In order to test the validity of our conclusions, we also employ the snowdrift game with the payoffs 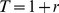, 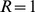, 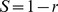 and 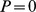, thus satisfying the ranking 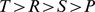, where 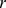 represents the so-called cost-to-benefit ratio. Indeed,the snowdrift game is frequently studied as an alternative to the perhaps better-known prisoner’s dilemma [34], [83].

### Definition of Reputation

Throughout the work, we assume that 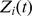 is the reputation of player 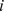 at time step 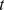. Each player possesses an identical reputation 1 before the game (*i.e.* 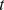 = 0). When the game proceeds, individual reputation at time step 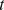 (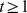) depends on the historical memory (namely, previous reputation situation) and present strategy selection, similar to the seminal idea of Fu *et al.* [74]. That is,
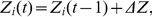
(6)where 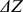 denotes the increment of reputation at time step *t*. If player *i* selects cooperation, 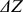 is 1, otherwise equalling to 0. It is obvious that individual reputation is evaluated in an accumulated way, which can get the illumination from some realistic situations. For example, a crucial index to estimate the success of a scientist is the total citation of his studies, in biological society individual study ability is usually based on the previous basic. Moreover, this definition of reputation is largely related to cooperating act, it becomes rather expectant that the established rule supports cooperation. In order to better carry out our research route, we will introduce individual reputation into strategy updating process.

### Strategy Updating

As for the interaction network, we use either a regular 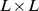 square lattice, a random regular graph (RRG) constructed as described in [76], or the small-world (SW) topology with an average degree of four generated via the Watts-Strogatz algorithm [84]. Each vertex *i* is initially designated as a cooperator (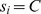) or defector (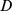) with equal probability. The game is iterated forward in accordance with the Monte Carlo simulation procedure comprising the following elementary steps. First, player 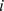 acquires its payoff 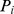 by playing the game with all its neighbors. Next, we evaluate in the same way the payoffs of all the neighbors of player 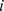. Lastly, it is also most important that player 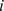 selects one neighbor 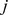 to update its strategy, which is closely related to the judgement of reputation.

Before the game each player is assigned with a parameter value 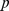 in the interval between zero and one to denote his inferring ability. This setting is performed uniformly irrespective of his initial strategy and remains unchanged during the simulations. Under such a case, we assume that players possess limited and different inferring ability to evaluate their opponents due to the cost and error of information dissemination. Therefore, neighbor having the highest reputation is selected with probability 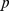, while randomly choosing one neighbor is left with the probability 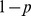. After the neighbor 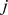 is chosen, player 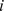 adopts the strategy 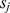 of the selected player 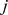 with the probability
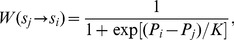
(7)where 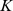 denotes the amplitude of noise or its inverse (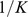) the so-called intensity of selection [51]. Besides the investigation of phase diagram we set 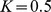, implying that better performing players are readily imitated, but it is not impossible to adopt the strategy of a player performing worse. During a full Monte Carlo step (MCS) all players have a chance to update their strategies once on average.

The results of Monte Carlo simulations presented bellow were obtained on populations comprising 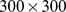 to 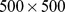 individuals, whereby the fraction of cooperators 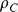 was determined within the last 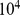 full steps of overall 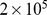 MCS. Moreover, since the random distributions of referring ability may introduce additional disturbances, the final results were averaged over up to 40 independent runs for each set of parameter values in order to assure suitable accuracy.
